# Epidemiology of Patients With Multiple Trauma and the Quality of Their Prehospital Respiration Management in Kashan, Iran: Six Months Assessment

**DOI:** 10.5812/atr.17150

**Published:** 2014-06-01

**Authors:** Mohsen Adib-Hajbaghery, Farzaneh Maghaminejad

**Affiliations:** 1 Trauma Nursing Research Center, Kashan University of Medical Sciences, Kashan, IR Iran; 2 Department of Medical Surgical Nursing, Faculty of Nursing and Midwifery, Kashan University of Medical Sciences, Kashan, IR Iran

**Keywords:** Quality of Health Care, Respiration, Prehospital Emergency Care, Multiple Trauma, Accident

## Abstract

**Background:**

Respiration management is an important and critical issue in prehospital transportation phase of multiple trauma patients. However, the quality of this important care has not been assessed in Iran Emergency Medical Services’ (EMS).

**Objectives:**

This study was conducted to investigate the quality of prehospital respiration management in patients with multiple trauma, referred to the Shahid Beheshti Trauma Center, Kashan, Iran.

**Patients and Methods:**

This cross-sectional study was conducted in the first six months of 2013. All the 400 patients with multiple trauma, transferred by EMS to the Shahid Beheshti Medical Center, were recruited. The study instrument was a checklist, which was completed through observation. Descriptive statistics were presented.

**Results:**

Out of all included individuals, 301 were males (75.2%) and 99 were females (24.8%). The most common mechanism of trauma was traffic accident (87.25%). Furthermore, 71.7% of the patients were injured in head and neck and chest areas. The quality of consciousness monitoring and airway management was desirable in 95% of the cases. However, the quality of monitoring patients’ respiration was only desirable in 42% of the cases. Only 18.6% of the patients received oxygen therapy during prehospital transportation.

**Conclusions:**

The quality of monitoring patients’ respiration and oxygen therapy was undesirable in most patients with multiple trauma. Therefore, the EMS workers should be retrained to apply proper respiration management in patients with multiple trauma.

## 1. Background

Trauma is a major public health problem and contributes a large burden of disability and suffering (1). Every day, almost 16,000 people die from various injuries around the world. Road traffic injuries are a major cause of mortality and accounts for more than 22% of all deaths, related to injuries (2) and would be the third leading cause of death worldwide by the year 2020 (3-5). Iran is a country with a high rate of road traffic crashes’ fatality and injury. According to statistics from the Forensic Medicine Organization of Iran, traffic crashes resulted in an annual average of 24,000 people (i.e. 3 persons per hour) expiration and approximately 240,000 injuries.(6, 7). Unfortunately, the problem is accelerating in developing countries due to rapid motorization and unsafe driving. In addition, while low-income and middle-income countries already account for more than 85% of all road traffic deaths in the world, the upsurge in the number of vehicles per inhabitant will lead to an anticipated 80% increase in injury mortality rates between 2000 and 2020 (3). In developing countries, most road traffic deaths happen during the prehospital phase (8). Consequently, prehospital trauma care (PHTC) and Emergency Medical Services (EMS) has received considerable attention during the past decades (9-11). This system not only transfers injured patients to the hospital setting, but also is responsible to provide them prehospital care, to stabilize their vital signs, to prevent additional injuries, death and disability (12, 13). A key pre-hospital measure has been provided to trauma patients is respiration management. During the prehospital phase, trauma patients need to be ventilated appropriately to preserve cellular metabolism and decrease the mortality rate (14-16). Good respiration management significantly decreases the mortality rate and improves patient outcomes after severe head injuries (17, 18). In recent years, the Iranian Ministry of Health and Medical Education have recognized the necessity of improving the quality of prehospital trauma care (19). Providing a high quality prehospital respiration management is an important intervention and evaluating the performance of the EMS in this area is an important component of assuring prehospital trauma care. However, to the best of our knowledge, the quality of prehospital respiration management is poorly known. A study in Occupied Palestine has shown that about 20% of patients with chest trauma or head injury did not received good respiration management in pre-hospital phase (20). A study on the quality of prehospital trauma care in Iran has also reported that 20% of trauma patients did not receive oxygen therapy in prehospital phase (21). Previously a report has been published on the quality of prehospital oxygen therapy in patients with multiple trauma (22). However, no study is available on the total respiration management in these patients.

## 2. Objectives

Considering the importance of respiration management in multiple trauma patients, this study aimed to investigate the quality of respiration management during prehospital transportation of these patients.

## 3. Patients and Methods

A cross-sectional study was conducted during the first six months of 2013. According to the information obtained from the archives of the prehospital EMS in Kashan, 350 cases of multiple trauma have been recorded in the same previous period. Then, the same number of the samples was estimated to be needed in this study. However, we recruited all patients with inclusion criteria consecutively and finally 400 multiple trauma patients were entered in the study. The study population was consisted of all patients with multiple trauma who had been transferred by EMS to the Trauma Center of Shahid Beheshti Medical Center, which is the main trauma center in Kashan city. The inclusion criteria were having multiple trauma, being alive at hospital admission, and being transferred to the trauma center by EMS. A patient with multiple trauma was defined as a patient having at least two or more injuries and these injuries put the patients at great need to emergency medical care (23). The study checklist was developed by the researchers through extensive literature review and counseling with trauma experts and consisted of three parts developed through in-depth literature review. The first part consisted of four questions regarding patients’ age, gender, job, and education level. The second part was a trauma assessment questionnaire (TAQ) that included 6-questions on the date of trauma occurrence, type of trauma (blunt, penetrating, or both), mechanism of trauma (traffic accident, fall, street attack, and debris fall), type of accident (pedestrian, bicycle, motorcycle, and car), time the trauma occurred (day or night) and the place the trauma occurred. The third part of the instrument consisted of 14 items for evaluation of respiration management during the prehospital transportation. This part consisted of two items on checking consciousness, 6 items on airway management, and 6 items on monitoring patients’ respiration. All items in respiration management scale (RMS) were scored on a three-point Likert scale in which two stood for ‘Done properly’, one for ‘Done improperly’, and 0 for either ‘Not done’ or ‘Not documented’. Accordingly, the total score of RMS ranged from zero to 28. Then, the total score was divided by 14 (the number of questions) to make a criteria for measuring the quality of respiration management. Consequently, scores lower and higher than two were interpreted as undesirable and desirable respiration management, respectively. To validate this assessment, the second researcher or a research assistant who were educated and tested for its correct performance were observed the patients’ respiration, orally asked patients: ‘did you receive oxygen in ambulance?’ and if a patient was unconscious, we checked the patient for being connected to oxygen administration devices such as nasal cannula or facemask.

Six nursing lecturers confirmed the content validity of the questionnaires and their comments were included in its final version. All the experts agreed that all items in the instrument are essential and also agreed on item relevance. The content validity ratio (CVR) and content validity index (CVI) of the instrument was equal to 1 (24, 25). To ensure the reliability of the instruments, two raters administered the instrument to ten patients. All data was collected by the second author and two co-researchers and at least one of them was present in the emergency department in each shift during the study period. The co-researchers were educated for data collection and tested for the correct data collection. Data were collected through observation, interviews with conscious patients and EMS staff and reviewing the patients’ records.

### 3.1. Data Analysis

Data analysis was performed using the SPSS software v.16.0 (SPSS Inc., Chicago, USA). No missing value was existed. Frequency and percent were presented for all variables and frequency tables were used. Also Chi-Square and Fisher's exact tests were used to test the relationship between the quality of prehospital respiration management and patients and trauma characteristics.

### 3.2. Ethical Considerations

This study was approved by the research ethics committee of Kashan University of Medical Sciences and issued by number 463 on May 4, 2013. Also, the study was granted by the university research deputy with grant number 9206. We explained the purposes of the study to the participants and ensured them of the confidentiality of their personal information. A verbal consent was obtained from the participants. Furthermore, data collection was done after permission of the hospital and unit authorities. We also observed all ethical issues in accordance with the last version of the Helsinki declaration.

## 4. Results

Among all patients, 301 were (75.2%) males. The patients ranged in age from two to 90 years and 175 (43.8%) were between 16 to 30 years old. Most of the patients were literate (75.3%) and 301 (25%) were industrial workers. In total, 150 (37.5%) of all the traumas had happened during holidays and 261 cases (65.2%) had happened in urban streets. The most common mechanism of trauma was traffic accident, which have occurred in 349 patients (87.25%). Motorcyclists were the majority of traffic accident victims (61.3%). Most of the trauma (273 cases, (68.3%)) had happened during daytime and 337 patients (84.2%) had experienced a mix of blunt and penetrating traumas, which were mainly (60%) on the head and neck area (Table 1). Table 2 shows that the quality of consciousness monitoring and airway management was desirable in 95% of all trauma cases. However, the quality of monitoring patients’ respiration was undesirable in 58% of the cases.

**Table 1.** Patients and Trauma Characteristics^a^

Variable	Results
**Age, y**	
16-30	175 (43.8)
31-45	95 (23.8)
46	92 (23.0)
**Level of education**	
Illiterate	99 (24.8)
Literate	301 (75.3)
**Participants’ occupation**	
Self-employment	94 (23.5)
Industrial worker	100 (25)
Homemaker	79 (19.75)
Student	56 (14)
Official worker	15 (3.75)
Other	56 (14)
**Mechanism of trauma**	
Traffic accident	349 (87.25)
Fall	37 (9.25)
Street attack	10 (2.5)
Debris fall	4 (1)
**Type of traffic accident**	
Pedestrian	52 (14.9)
Automobile passenger	83 (23.8)
Motorcyclist	214 (61.3)
**Type of trauma**	
Penetrating	43 (10.8)
Blunt	20 (5.0)
Mixed	337 (84.2)
**Site of injury**	
Head and neck	210 (60.00)
Upper limb	228 (65.14)
Chest	41 (11.71)
Abdomen, back, and pelvis	113 (32.28)
Lower limb	213 (60.85)
**Day of Trauma**	
Weekend and holidays	150 (37.5)
Usual days	250 (62.5)
**Time of trauma**	
Day	273 (68.3)
Night	127 (31.8)
**Place the Trauma Occurred**	
Home	10 (2.5)
Workplace	13 (3.3)
Urban streets	261 (65.2)
Country roads	116 (29)

^a^Data are presented as No. (%).

**Table 2.** The Quality of Respiration Management in Patients With Multiple Trauma^a^

The Variable Evaluated	Results
**Total quality**	
Desirable	380 (95.0)
Undesirable	20 (5.0)
**Monitoring the consciousness**	
Desirable	381 (95.2)
Undesirable	19 (4.8)
**Airway management**	
Desirable	380 (95)
Undesirable	20 (5)
**Monitoring patients’ respiration**	
Desirable	168 (42)
Undesirable	232 (58)

^a^Data are presented as No. (%).

In total, 236 patients needed oxygen therapy during the prehospital phase; however, only 44 of them (18.64%) had received oxygen. None of the patients was assessed regarding oxygen saturation. Moreover, lung auscultation and rhythm of respiration were not documented in patients’ prehospital medical records. On the other hand, in 72 patients (30.50%) whose prehospital medical records indicated the administration of oxygen, reported that they have not received oxygen therapy. Moreover, in 50.84% of all cases, oxygen therapy had not been documented at all (Table 3). No significant statistical relationship was observed between the quality of prehospital respiration management and characteristics of patients and traumas (Table 4).

**Table 3.** Documentation of Pre-Hospital Oxygen Therapy^a^

Oxygen Therapy	Results
**Not performed**	
Documented	72 (30.5)
Not documented	120 (50.9)
**Documented and performed**	44 (18.6)
**Total**	236 (100)

^a^Data are presented as No. (%).

**Table 4.** The Quality of Pre-Hospital Respiration Management According to Patients and Trauma Characteristics^a^

	Quality Respiration Management		P Value
	Desirable	Undesirable	
**Gender**			0.792^b^
Female	95 (96)	4 (4)	
Male	285 (94)	16 (5.3)	
**Age, y**			0.354^b^
< 15	34 (89.5)	4 (10.5)	
16-30	167 (95.4)	8 (4.6)	
31-45	92 (96.8)	3 (3.2)	
46	88 (95.7)	4 (4.3)	
**Level of education**			0.371^b^
Illiterate	93 (93.9)	6 (6.1)	
Literate	287 (95.3)	14 (4.7)	
**Location of the accident**			0.198^c^
Home and workplace	23 (100)	0	
Urban streets	250 (95.8)	11 (4.2)	
Country roads	107 (92.2)	9 (7.8)	
**Type of trauma**			0.787^b^
Penetrating	42 (97.7)	1 (2.3)	
Blunt	19 (95)	1 (5)	
Mixed	319 (94.7)	18 (5.3)	
**Mechanism of trauma**			0.761^c^
Traffic accident	331 (94.8)	18 (5.2)	
Non Traffic accident	49 (96.1)	2 (3.9)	
**Day of trauma**			0.236^c^
Weekend and holidays	145 (96.7)	5 (3.3)	
Usual days	235 (93.9)	15 (6.1)	
**Time of trauma**			0.068^c^
Day	263 (96.4)	10 (3.6)	
Night	116 (92.1)	10 (7.9)	
**Type of traffic accident**			0.650^b^
Pedestrian	48 (92.3)	4 (7.7)	
Automobile passenger	79 (95.2)	4 (4.8)	
Motorcyclist	204 (95.3)	10 (4.7)	

^a^Data are presented as No. (%).

^b^Fisher's exact test.

^c^Chi-square.

## 5. Discussion

The aim of this study was to investigate the quality of prehospital respiration management in patients with multiple trauma. The study showed that most of patients with multiple trauma were males between 16 to 30 years old. In addition, the most common mechanism of trauma was road traffic accidents and such events were more common among motorcyclists. This is consistent with previous studies on the epidemiology of traumas (26-30). Nguyen et al*.* (26), and Lin et al*.* (27) also reported that the most common mechanism of trauma was accidents. However, Lin et al*.* reported that motorcycle accidents were the third leading cause of traumas (27). On the other hand, Engel et al*.* and Janssen and Burns found that the most common mechanism of trauma was falling down from heights (28, 29). Factors such as extensive use of motorcycles in Iran - particularly by unlicensed young motorcyclists - and their reluctance over using safety helmet contribute to the high prevalence of motorcycle accidents. The current study showed that about two third of the multiple trauma patients were injured at head and neck and chest. According to Barsuk et al*.* (20) and the protocols of EMS in Iran, all multiple trauma patients with head and chest injuries need to receive complementary oxygen during the prehospital phase. However, our findings showed that though the quality of consciousness monitoring and airway management was desirable in more than 95% of the multiple trauma patients, the quality of monitoring patients’ respiration was undesirable in most cases and oxygen had been administered only to 18.64% of patients who needed it. The rate of oxygen administration was about 80% in studies conducted by Barsuk et al*.* (20) and Ahmadi-Amoli et al. (21). Such findings show that the EMS workers are not fully aware of the importance of respiration monitoring and oxygen administration in multiple trauma patients. Even if they manage the airway appropriately, they do not observe the respiration and oxygen demands properly and did not monitor the blood oxygen saturation despite that all ambulances are equipped with oxygen saturation monitoring devices. Lack of an effective in-service education, supervision, and evaluation for the EMS staff might be contributed to these findings. Haghparast Bidgoli et al*. *in two different studies (11, 31) have reported that paramedics’ lack of knowledge, experience, and mastery - secondary to lack of continuing education programs - negatively affects the quality of pre-hospital patient care. Therefore, development and implementation of effective in-service education programs in the area of respiration management and oxygen therapy are strongly recommended.

In the current study, no significant relationship was found between the quality of prehospital respiration management and characteristics of patients and traumas. However, a surprising finding of the present study was that for about one third of patients who needed oxygen, the administration of oxygen had been documented in their pre-hospital medical records but did not actually administered. Haghparast Bidgoli et al*.* have reported that as paramedics are paid poorly, they feel compelled to work in double or long shifts that in turn sacrifice the quality of the care (31). Given the importance of prehospital oxygen therapy and prevalence of road traffic accidents in our country, careful supervision and evaluation of prehospital care providers seems crucial. Although the consciousness monitoring and airway management was desirable, the quality of monitoring patients’ respiration and oxygen administration was undesirable in most patients with multiple trauma. Therefore, the EMS workers should be retrained to apply proper respiration management in these patients. All data collection efforts and observations in this study were conducted by the second author and this has decreased the possibility of inter-rater variations. However, the study was only conducted in 6 months, and in one center. Therefore, the data may not necessarily mirror the performance of all EMS staff countrywide and further studies with longer duration are suggested to be conducted in different areas of the country. Also, studies are needed to assess the barriers of standard performance in EMS.
